# Antityrosinase activity of *Euphorbia characias* extracts

**DOI:** 10.7717/peerj.1305

**Published:** 2015-10-13

**Authors:** Francesca Pintus, Delia Spanò, Angela Corona, Rosaria Medda

**Affiliations:** Department of Sciences of Life and Environment, University of Cagliari, Monserrato, Cagliari, Italy

**Keywords:** B16F10 melanoma cells, *Euphorbia characias*, Melanogenesis, Tyrosinase inhibitors

## Abstract

Tyrosinase is a well-known key enzyme in melanin biosynthesis and its inhibitors have become increasingly important because of their potential use as hypopigmenting agents. In the present study, the anti-melanogenic effect of aqueous and ethanolic extracts from *Euphorbia characias* leaves, stems, and flowers in cell-free and cellular systems was examined. All the extracts showed inhibitory effects against mushroom tyrosinase with leaf extracts exhibiting the lowest IC_50_ values of 24 and 97 µg/mL for aqueous and ethanolic extracts respectively. Enzyme kinetic analysis indicated that leaf aqueous extract acts as a mixed type inhibitor, while ethanolic extract shows a competitive inhibition effect on mushroom tyrosinase using L-DOPA as substrate. In addition, the inhibitory effect of leaf extracts on tyrosinase activity and melanin production was examined in murine melanoma B16F10 cells. Cellular tyrosinase activity as well as levels of melanin synthesis are reduced in a dose-dependent manner by extracts in cells treated with *α*-melanocyte stimulating hormone (*α*-MSH). The effects are comparable, and sometimes even better, than that of kojic acid, a well known tyrosinase inhibitor used for reference. All these results suggest that *E. characias* could be a great source of the natural inhibitors from tyrosinase and has the potential to be used as a whitening agent in therapeutic fields.

## Introduction

Melanogenesis is a physiological process resulting in the synthesis of melanin pigments which are responsible for skin pigmentation and provide a beneficial effect in preventing skin damage under normal condition. Melanin is a biopolymer synthesized by melanocytes within specialized organelles called melanosomes and then secreted and distributed in the basal layer of the dermis. It plays a crucial role in preventing UV-induced skin damage by absorbing UV sunlight and removing reactive oxygen species. Tyrosinase (EC 1.14.18.1) is the rate-limiting enzyme involved in melanin synthesis. It is a copper-containing enzyme that catalyzes the hydroxylation of tyrosine (monophenolase activity) and the oxidation of 3,4-dihydroxyphenylalanine (l-DOPA) to o-dopaquinone (diphenolase activity). Oxidative polymerization of dopaquinone derivatives gives rise to melanin (Parvez et al., 2006). Despite its advantages, abnormal production or distribution of melanin is the cause of various dermatological disorders such as melasma, lentigines, age spots and post-inflammatory hyperpigmentation. Due to the key role of tyrosinase in melanin pathway, its inhibitors have become increasingly important for medicinal and cosmetic products that may be used as powerful skin-whitening agents for treating skin disorders (Kim & Uyama, 2005).

Tyrosinase is also responsible for the undesired enzymatic browning of fruits and vegetables because it catalyzes the oxidation of phenolic compounds to the corresponding highly active quinones which lead to the formation of brown polymeric pigments. These quinones may irreversibly react with amino or sulfhydryl groups of proteins, destroying essential amino acids and reducing proteins digestibility and their nutritive value. In order to prevent the browning and to preserve the nutritional value of food, development of good tyrosinase inhibitors has great importance for the agricultural field and the food industry (Wang et al., 2011).

Most of the inhibitors so far characterized are synthetic compounds or derived from higher plants such as polyphenols, flavonoids, aldehydes and their derivatives (Loizzo, Tundis & Manichini, 2012). Among synthetic compounds, the specificity and mechanism of inhibition (competitive, non-competitive or mixed) are extremely variable and characteristic of the chemical nature of the compounds (Ha et al., 2012). Some inhibitors affect both mono- and diphenolase activities of tyrosinase by chelating the active site of the enzyme. Moreover, some compounds from fungal sources have also been identified for their inhibitor activity. For example, kojic acid, a fungal metabolite, is the most intensively studied inhibitor of tyrosinase and is often used as the positive control in the literature for comparing the inhibitory strength of the finding inhibitors.

Since plants are a rich source of bioactive chemicals that are mostly free from harmful side effects, interest in finding tyrosinase inhibitors from natural sources is increasing (Oh et al., 2011; Chan, Kim & Cheah, 2011; Rashed et al., in press). The effects of plant materials could result from isolated substances but typically derive from the synergy of different bioactive compounds present in the plant.

**Figure 1:**
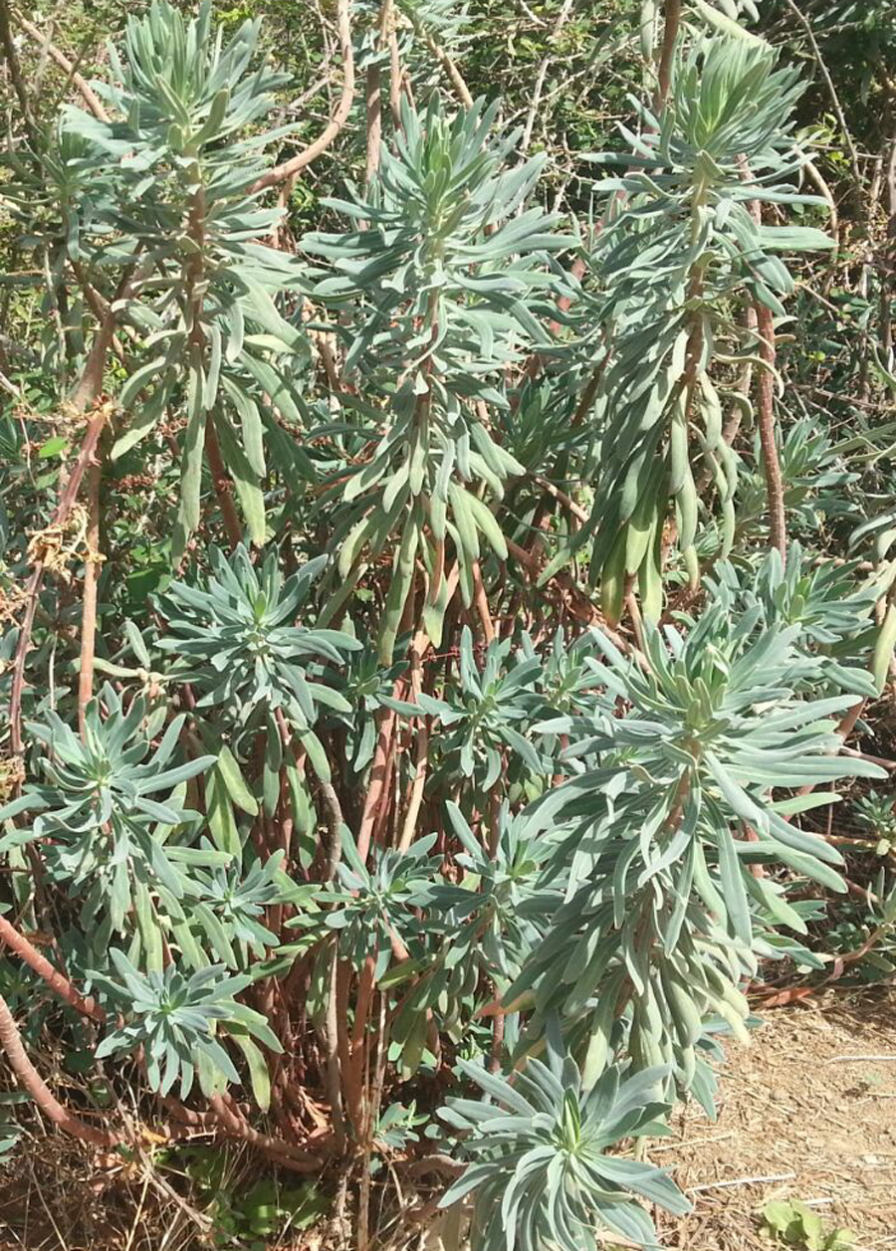
*Euphorbia characias*. The Mediterranean shrub *Euphorbia characias* subsp. *characias* from southern Sardinia (Italy).

*Euphorbia characias* (Fig. 1) is a typical shrub commonly occurring in various habitats (rocky hillsides, along road verges, in open woods and in olive groves) in vast areas of the Mediterranean basin. A milky latex is characteristic of the Euphorbiaceae family, and seems to have a key role in plant defense mechanisms by repelling and killing phytopathogens, and sealing wounded areas (Pintus et al., 2010).

*E. characias* latex has been studied with regard to its antioxidant and non-protein components (Pintus et al., 2013) but several proteins have been isolated and deeply characterized (Mura et al., 2008; Pintus et al., 2009; Pintus et al., 2011; Dainese et al., 2014; Spanò et al., in press). It has also been identified in *E. characias*, a *cis*-prenyl transferase (Spanò et al., 2015) which is the enzyme responsible of the synthesis of the natural rubber occurring in the plant latex (Spanò et al., 2012).

Considering the attention centered on the plant latex, up to now very little attention has been paid to other parts of the plant (Palomino-Schätzlein et al., 2011).

In the present study, we describe the antimelanogenesis activity of different extracts from leaves, stems and flowers of *E. characias*. The ability of *E. characias* extracts to inhibit tyrosinase activity was evaluate using cell-free mushroom tyrosinase and a cellular system with B16F10 mouse melanoma cells.

## Materials and Methods

### Reagents

All chemical were obtained as pure commercial products from Sigma Chemical Co (St. Louis, Missouri, USA) and used without further purification.

### Spectrophotometry

Absorption spectra and data from activity assays were recorded with an Ultrospec 2100 spectrophotometer (Biochrom Ltd., Cambridge, England) using cells with a 1 cm path length.

### Sample preparation

*E. characias* subsp. *characias* was identified by Prof. Annalena Cogoni, Department of Science of Life and Environment, Section of Botany and Botanical Garden, University of Cagliari, Italy. Leaves, stems and flowers of *E. characias* were collected at several locations in southern Sardinia (Italy), immediately frozen at −80 °C and then lyophilized. The lyophilized plant materials (1 g) were extracted in 10 mL of water (AE, aqueous extract) or in ethanol (EE, ethanol extract) for 24 h at room temperature under continuous stirring. After diluting ethanol extracts 10-fold with water, all extracts were then lyophilized. Dried powers (1 mg) were dissolved in 1 mL of the apposite solvent (water or 10% ethanol:water for AE and EE respectively) before use.

### Mushroom tyrosinase activity

The inhibitory effect of *E. characias* extracts on tyrosinase activity was determined spectrophotometrically with the degree of inhibition of mushroom tyrosinase-catalysed oxidation of l-DOPA. The reaction mixture contained 25 mM phosphate buffer (pH 6.8) with or without samples and l-DOPA (1.25 mM). Then, tyrosinase (100 U/mL) was added into the mixture and the activity was determined by following the increase in absorbance at 475 nm resulting from the formation of the dopachrome product. The inhibition of tyrosinase activity was calculated with the following formula: Inhibition (%) = [1 − (A_475_ in sample/A_475_ in control)] × 100%.

In order to determine the mode of inhibition, assays were also performed at different concentration ofl-DOPA (0.25–1.25 mM) and extracts (0–0.25 mg/mL). Kinetics data were analysed using the Lineweaver-Burk plot.

### Determination of copper chelation

The copper chelating capacity of the extracts was determined by the UV/Vis spectra according to Kubo et al. (2000). Extracts (∼20 µg/mL) were mixed with different concentration of CuSO_4_ (0.01–1.5 mM) and, after incubation at 25 °C for 10 min, absorption spectra from 200 to 800 nm were recorded.

### Cell culture

Murine melanoma B16F10 cells (CRL-6475) were purchased from the American Type Culture Collection (ATCC, Manassas, Virginia, USA). The cells were cultured in Dulbecco’s Modified Eagle Medium (DMEM) containing 10% fetal bovine serum (FBS; Gibco, NY, USA), and 1% penicillin/streptomycin at 37 °C in a humidified atmosphere with 5% CO_2_.

Cell viability was detected by the colorimetric 3-(4,5-dimethylthiazol-2-yl)-2,5-diphenyltetrazolium bromide (MTT) assay (Mosmann, 1983). Briefly, cells were seeded in a 96-well plate (10^4^ cells/well) and incubated with samples at different concentration (0.05–0.5 mg/mL). After 48 h of incubation, cells were labelled with MTT solution for 3 h at 37 °C. The resulting violet formazan precipitates were dissolved in isopropanol and the absorbance of each well was determined at 590 nm using a microplate reader with a 630 nm reference.

### *α*-MSH treatment

B16F10 cells were seeded at a density of 10^5^ cells/mL in 6-well plates containing 10% fetal bovine serum and 1% penicillin/streptomycin at 37 °C in a humidified atmosphere with 5% CO_2_. After 24 h, the medium was substituted by fresh one supplemented with 100 nM *α*-MSH and different concentration of plant extracts (0.05–0.5 mg/mL) and incubated for 48 h. Cells treated with 100 nM *α*-MSH and kojic acid were used as positive control and for comparing the inhibitory strength of the finding *E. characias* inhibitors. All experiments reported in next sections were performed with these stimulated-cells by using the above procedure.

### Intracellular tyrosinase activity

*α*-MSH-stimulated cells were plated in 60*π*-dishes at a density of 10^5^ cells/mL and incubated for 48 h in absence or presence of samples (0.05–0.25 mg/mL). The cells were washed with PBS and lysed in 50 mM phosphate buffer (pH 6.8) containing 1% Triton X-100 and 0.1 mM phenylmethyl-sulfonyl fluoride. Cellular lysates were centrifuged at 12,000 rpm for 20 min at 4 °C. The supernatant was collected and the protein content was determined by the Bradford method using BSA as standard (Bradford, 1976). The cellular extract was incubated with l-DOPA (1.25 mM) in 25 mM phosphate buffer (pH 6.8) and the absorbance at 475 nm was read until the reaction has finished.

### Melanin content assay

*α*-MSH-stimulated cells were plated on 60*π*-dishes at a density of 10^5^ cells/mL and incubated for 48 h in absence or presence of samples (0.05–0.25 mg/mL). After washing with PBS, cells were harvested and an aliquot was used for protein quantification while the remaining cells were centrifuged and lysed with NaOH 1 M at 100 °C for 1 h. Melanin concentrations were calculated by comparison of the absorbance at 405 nm using a standard curve of synthetic melanin.

### l-DOPA staining assay

The DOPA-staining assay was performed as reported by Sato and other authors with some modifications (Sato et al., 2008). Cells were treated for 48 h with *α*-MSH alone or *α*-MSH plus leaves extracts at different concentration or kojic acid at 100 or 200 µM as positive control. After treatment, cells were harvested with lysis buffer, as described in ‘Intracellular tyrosinase activity’. Protein extracts (5 µg) were then mixed with 10 mM Tris–HCl buffer, pH 7.0, containing 1% SDS, without mercaptoethanol or heating, and resolved by 8% SDS-polyacrylamide gel electrophoresis. After running, gel was rinsed in 0.1 M phosphate buffer (pH 6.8) and equilibrated for 30 min twice. The gel was then transferred in a staining solution containing 0.1 M phosphate buffer (pH 6.8) with 5 mM l-DOPA and incubated in the dark for 1 h at 37 °C. Tyrosinase activity was visualized in the gel as dark melanin-containing bands.

### Statistical analysis

Data are expressed as mean ± SD from three independent experiments. The statistical analysis of differences between various treatments was determined by the Student’s *t*-test. Values of *p* < 0.05 were considered statistically significant. Statistical analysis was performed with GraphPad Prism 6 software (GraphPad Software, San Diego, California, USA).

## Results and Discussion

### Inhibition of mushroom tyrosinase activity by *E. characias* extracts

**Figure 2:**
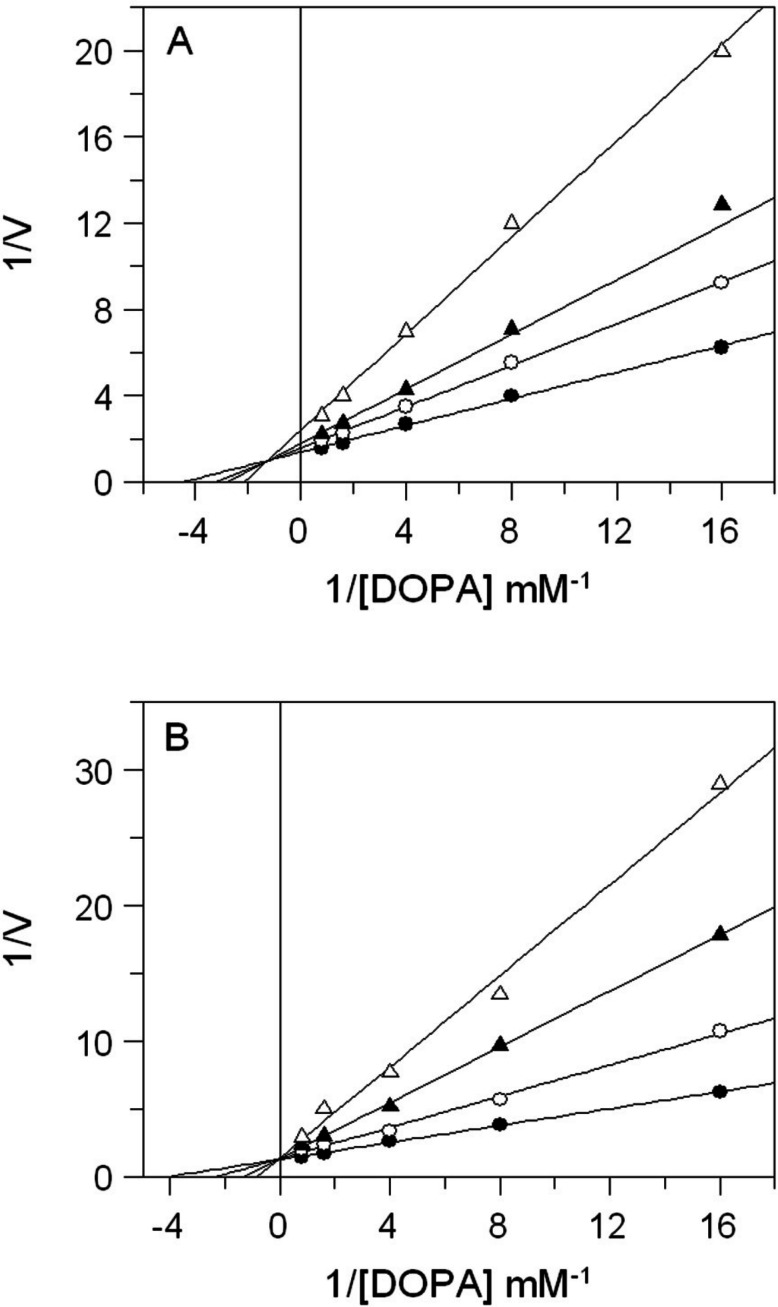
Inhibition of tyrosinase activity by *E. characias* leaves extracts. Lineweaver-Burk plots for inhibition of extracts on mushroom tyrosinase activity using l-DOPA as substrate. Reaction mixtures (1 mL) contained mushroom tyrosinase in 25 mM phosphate buffer (pH 6.8) and l-DOPA and extracts at different concentrations. (A) Aqueous extract: the concentration used were 0 (●), 0.05 mg/mL (○), 0.1 mg/mL (▴) and 0.25 mg/mL (▵). (B) Ethanolic extract: the concentration used were 0 (●), 0.05 mg/mL (○), 0.1 mg/mL (▴) and 0.15 mg/mL (▵).

The effects of *E. characias* extracts on mushroom tyrosinase activity using L-DOPA as substrate, are reported in Table 1. The results show that all extracts have a direct inhibitory activity against mushroom tyrosinase, with aqueous and ethanolic extracts from leaves (AEL and EEL respectively) exhibiting the stronger inhibitory effect. In fact, AEL shows an IC_50_ of 0.12 mg/mL, which is about 15- and 4-fold lower than IC_50_ values of stems and flowers aqueous extracts (1.8 and 0.49 mg/mL respectively). Effect of EEL is even better, with an IC_50_ value of 34 µg/mL being 32- and 4-fold lower than IC_50_ of stems and flowers ethanolic extracts (1.1 and 0.15 mg/mL respectively). Kojic acid, used as standard tyrosinase inhibitor, is obviously a more potent inhibitor. It is not surprising as it is a single molecule, whereas plant extracts are a mixtures of numerous compounds. Thus, the real concentration of active compond is lower than the IC_50_ value. The results look better than other plants extracts (Wang et al., 2011; Jo et al., 2012) and *E. characias* leaves extracts seem to be even more efficient and could be a promising sources of tyrosinase inhibitors. We have therefore focused our attention on extracts from leaves that show the best enzyme inhibition. The kinetic behaviour of tyrosinase at different concentration of l-DOPA and AEL or EEL was investigated. The mode of inhibition of the enzyme was determined by Lineweaver-Burk plot analysis, as shown in Fig. 2. Kinetic analysis suggests that AEL acts as a mixed-type inhibitor since increasing the concentration of extract resulted in a family of straight lines with different slope and intercept, which intersected in the second quadrant (Fig. 2A). In this case, the inhibitor can bind not only with the free enzyme but also reduce the affinity of the substrate, whereas it did not bind to the active site of the enzyme. The equilibrium constants for binding with the free enzyme (*K_i_*) and with the enzyme–substrate complex (${K}_{i}^{{\prime}}$) were obtained from the slope or the 1/*V*_max_ values (*y*-intercepts) versus inhibitor concentration, respectively. The values of *K_i_* and *K_i_*’ of AEL were determined to be 0.097 and 0.33 mg/mL, respectively.

**Table 1:** Inhibition of tyrosinase by *E. characias* extracts. Effect of aqueous and ethanolic extracts of leaves, stems and flowers of *E. characias* is expressed as IC_50_ values. Kojic acid is reported as standard inhibitor.

Part of plant	Extract	IC_50_ (mg/mL)
Leaves	Aqueous	0.12 ± 0.010
	Ethanol	0.034 ± 0.002
Stems	Aqueous	1.8 ± 0.13
	Ethanol	1.1 ± 0.09
Flowers	Aqueous	0.49 ± 0.025
	Ethanol	0.15 ± 0.011
Kojic acid		(0.8 ± 0.03) × 10^−3^

The inhibitory mechanism of EEL on mushroom tyrosinase is reported in Fig. 2B. In this case, increasing the concentration of extract, *K_m_* increased but *V*_max_ remained the same as common value in the *y*-axis, suggesting that EEL worked as competitive inhibitor of tyrosinase activity. An inhibition constant (*K_i_*) of 23.7 µg/mL was obtained from the secondary plot of the slope (*K_m_*/*V*_max_) versus EEL concentration.

Given that tyrosinase is a copper-containing enzyme, many compounds inhibit the activity of tyrosinase by chelating copper that plays a key role in the active site of the enzyme. Thus, we evaluated the copper chelating capacity of leaves extracts by spectra analysis.

**Figure 3:**
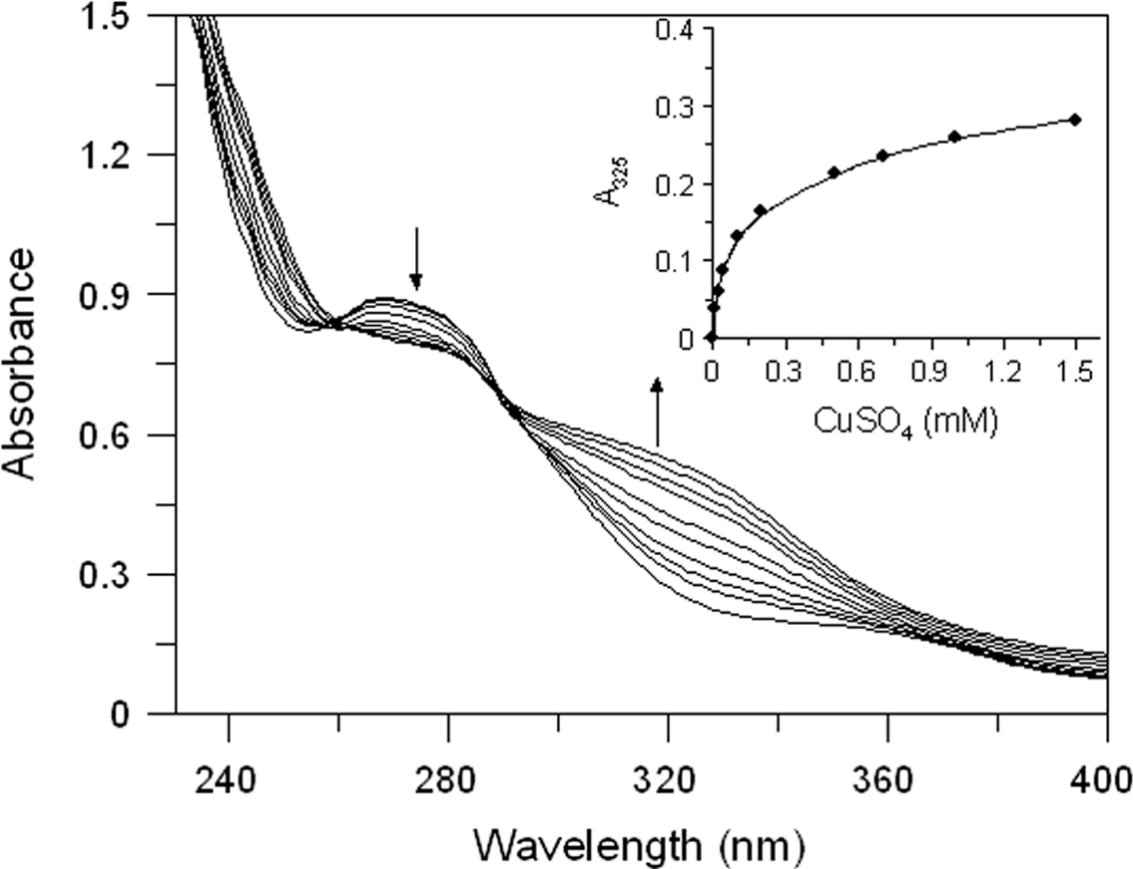
Effect of copper ions on *E. characias* ethanolic leaves extract. Absorption spectra of EEL in presence of various concentration of copper ions. Arrows indicate the decrease in absorbance at 265 nm and the contemporary increase in absorbance at 325 nm, after CuSO_4_ addition. Inset: increase of absorbance at 325 nm vs CuSO_4_ concentation.

Both extracts have proved to be able to bind copper ion. In Fig. 3 we reported the absorption spectrum of EEL (20 µg/mL) that exhibits a major peak at 265 nm. After addition of an excess of Cu^2+^, a characteristic bathochromic shift (from 265 to 325 nm) were observed and the increase of absorbance at 325 nm was positively related to the CuSO_4_ concentration (Fig. 3 inset). The addition of increasing doses of Cu^2+^ (0.01–1.5 mM) caused a gradual decrease in the magnitude of absorbance at 265 nm with the concomitant formation of the new peak at 325 nm. It showed a copper chelating capacity of EEL in a dose-dependent manner. These findings revealed that EEL inhibits tyrosinase activity in a competitive manner and this may be comes from its ability to chelate copper at the active site of the enzyme.

### Effect of *E. characias* extracts in cell culture system

**Figure 4:**
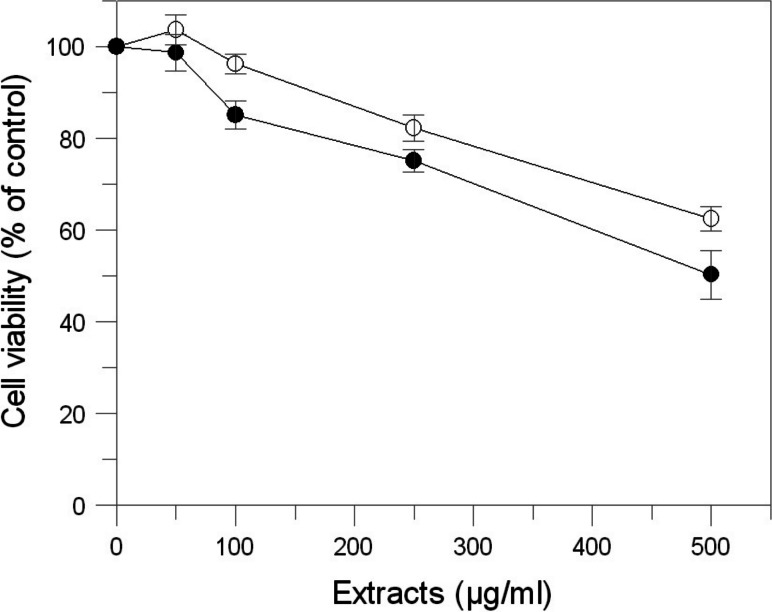
Effect of *E. characias* extracts on cell viability in B16F10 melanoma cells. After 48 h incubation with aqueous (○) or ethanolic (●) extracts, cell viability was determined by MTT assay. Data are expressed as mean ± SD from three independent experiments.

The effects of *E. characias* leaves extracts on cell viability, cellular tyrosinase and melanin level in B16F10 melanoma cells were determined. In order to determine the safety of these extracts, cells were treated with various concentration of samples for 48 h and were examined using MTT test. The results indicate that AEL and EEL are no considerable cytotoxic in B16F10 melanoma cells (Fig. 4). Cell viability was still about 85% at the concentration of 250 µg/mL for AEL and 100 µg/mL for EEL, so we performed further experiments using up to these extracts concentrations.

**Figure 5:**
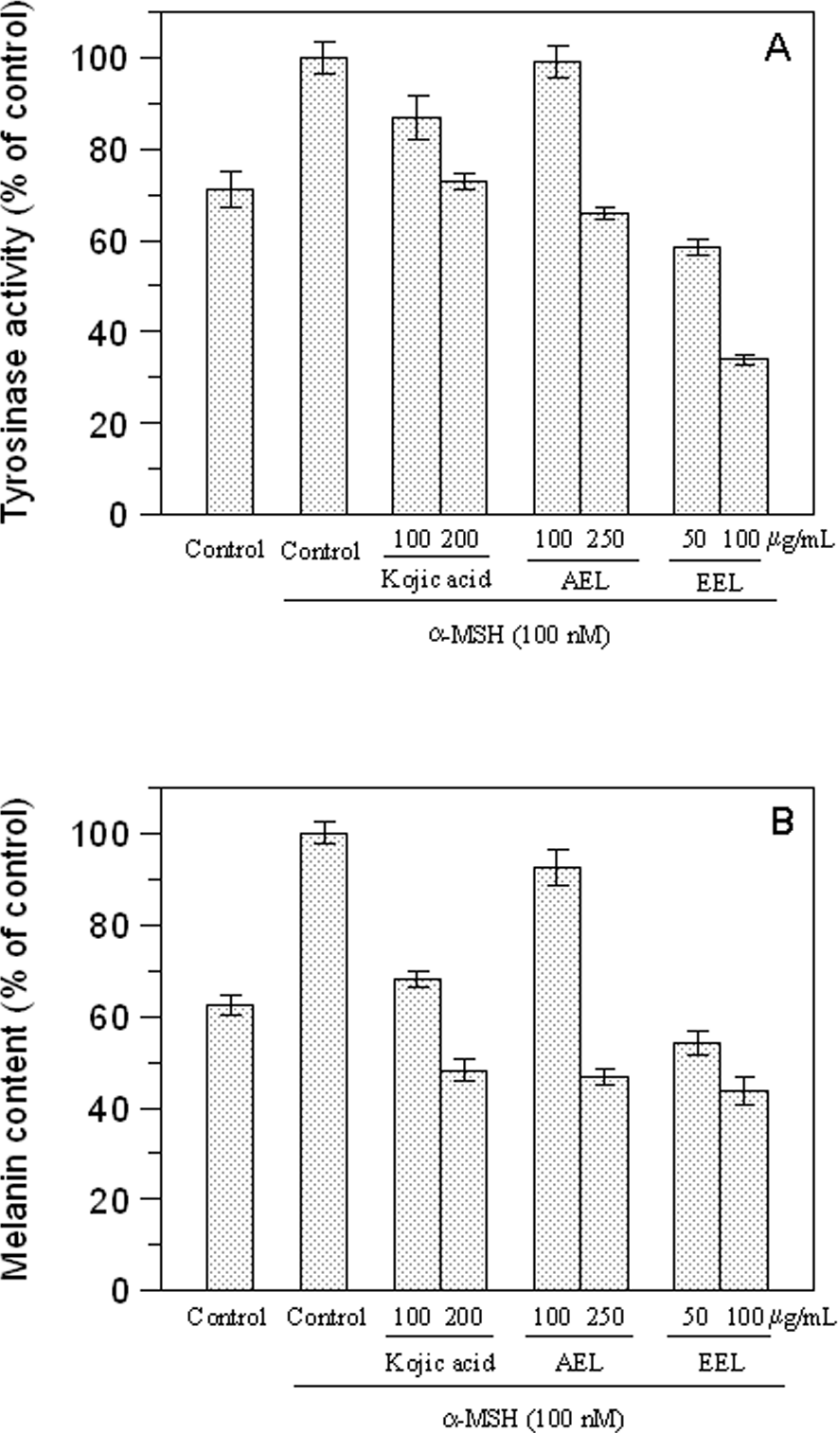
Effect of leaves extracts on B16F10 melanoma cells. Tyrosinase activity (A) and melanin production (B) are expressed as percentage of the control and the effects of extracts were compared with kojic acid as standard inhibitor.

We examined the inhibitory effect of the extracts on the tyrosinase activity of B16F10 cells treated with 100 nM *α*-MSH. Upon exposure to *α*-MSH alone, the tyrosinase activity was significantly increased, compared to untreated cells (Fig. 5A). After 48 h incubation with leaves extracts, tyrosinase activities were 99% (*p* > 0.05) and 66% (*p* < 0.05) at 100 and 250 µg/mL of AEL and 58.5% and 34% at 50 and 100 µg/mL of EEL (*p* < 0.05). Thus, AEL and EEL significantly reduced the tyrosinase activity in murine cells in a dose-dependent manner. The inhibitory effects of ethanolic extract was even much stronger than that of kojic acid, the positive control, that shows a tyrosinase activity of 86.6% and 73% at 100 and 200 µg/mL respectively.

We also evaluated the melanin content of B16F10 cells in the presence of extracts and Fig. 5B shows that also melanin synthesis was inhibited in a dose-dependent manner. The aqueous extract revealed, also in this case, to be almost ineffective at the concentration of 100 µg/mL (*p* > 0.05) but 51.8% of inhibition was obtained at 250 µg/mL (*p* < 0.05). Comparing the extracts and kojic acid at the same concentration as 100 µg/mL, EEL exerted the higher cellular melanogenesis effect with an inhibition of 56.2%, being higher than that of kojic acid (31.8%) and AEL (7.4%).

**Figure 6:**
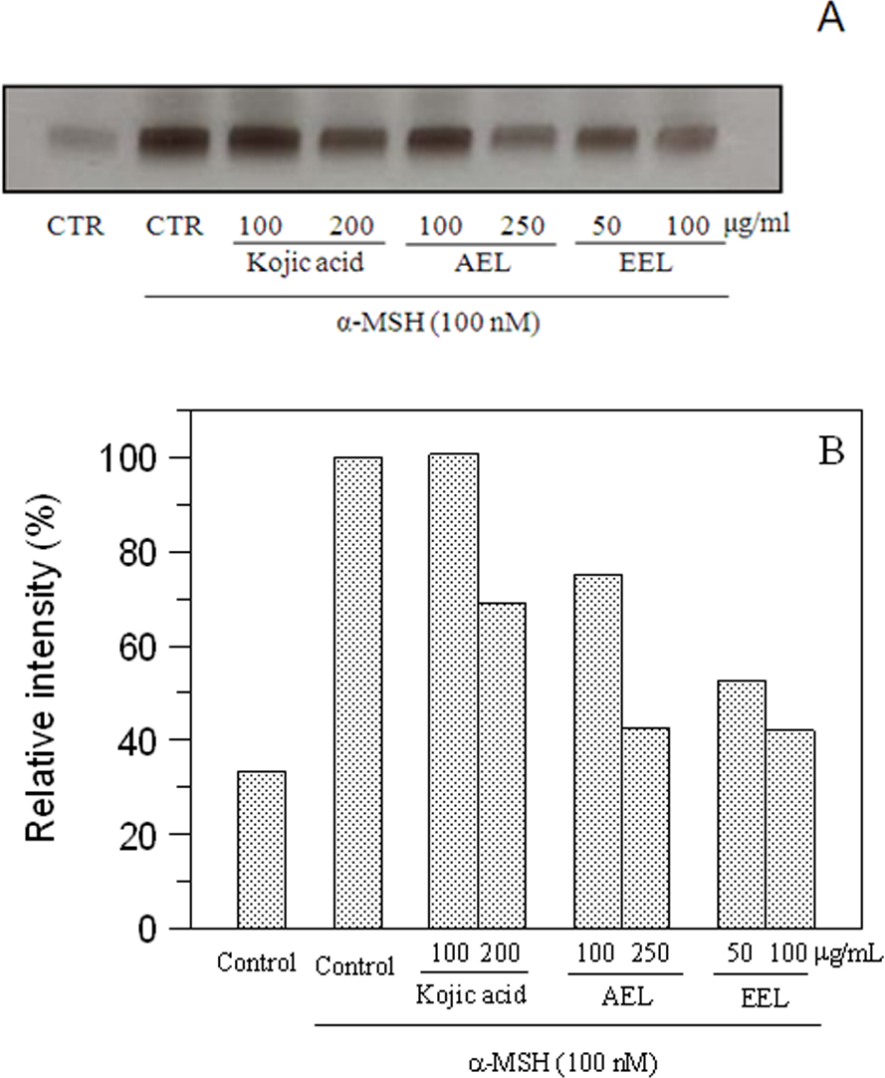
Effect of leaves extracts on B16F10 cells by L-DOPA staining. Tyrosinase activity was estimated by zymography (A) and the relative intensity of bands was determined with ImageJ software (B).

The inhibitory effect of melanogenesis by *E. characias* extracts was confirmed through the results of tyrosinase zymography. DOPA staining assay was carried out with lysates of *α*-MSH-stimulated B16F10 cells treated with or without extracts (Fig. 6). Treatment with *α*-MSH created a dark band compared to that of untreated control. Upon incubation with extracts, activity of tyrosinase decrease and lighter bands were observed. A lower intensity of the bands means a greater effect of inhibition. The results confirmed the inhibitory effects of the extracts.

In conclusion, we demonstrate that extracts from *Euphorbia characias* are effective in inhibiting tyrosinase activity using mushroom tyrosinase enzymes and in the cellular system. Leaf extracts have the most potent effect and inhibit tyrosinase activity and melanin synthesis in B16F10 melanoma cells, without cytotoxicity. Ethanolic extract causes a strong inhibition of melanogenesis even higher than kojic acid, the standard inhibitor.

Experiments to fractionate the extracts and to isolate the single active components responsible for the inhibition activity are now underway. These molecules might be useful in the food industry as antibrowning agents or in the medical field to treat hyperpigmentation disorders.

## Supplemental Information

10.7717/peerj.1305/supp-1 Supplemental Information 1 RawData_Figure_2

10.7717/peerj.1305/supp-2 Supplemental Information 2 RawData_Figure_3

10.7717/peerj.1305/supp-3 Supplemental Information 3 RawData_Figure_4

10.7717/peerj.1305/supp-4 Supplemental Information 4 RawData_Figure_5

10.7717/peerj.1305/supp-5 Supplemental Information 5 RawData_Figure_6
